# Lessons learned from COVID-19 modelling efforts for policy decision-making in lower- and middle-income countries

**DOI:** 10.1136/bmjgh-2024-015247

**Published:** 2024-11-08

**Authors:** Collins J Owek, Fatuma Hassan Guleid, Justinah Maluni, Joyline Jepkosgei, Vincent O Were, So Yoon Sim, Raymond Cw Hutubessy, Brittany L Hagedorn, Jacinta Nzinga, Jacquie Oliwa

**Affiliations:** 1 Department of Public and Global Health, University of Nairobi, Nairobi, Kenya; 2 KEMRI-Wellcome Trust Research Programme Nairobi, Nairobi, Kenya; 3 Data Synergy and Evaluation Unit, African Population and Health Research Center, Nairobi, Kenya; 4 World Health Organization, Geneva, Switzerland; 5 Institute for Disease Modelling, Bill & Melinda Gates Foundation, Seattle, Washington, USA; 6 Health Economics Research Unit, KEMRI-Wellcome Trust Research Programme, Nairobi, Kenya; 7 Health Services Unit, KEMRI-Wellcome Trust Research Programme, Nairobi, Kenya

**Keywords:** COVID-19, Mathematical modelling, Decision Making

## Abstract

**Introduction:**

The COVID-19 pandemic had devastating health and socioeconomic effects, partly due to policy decisions to mitigate them. Little evidence exists of approaches that guided decisions in settings with limited pre-pandemic modelling capacity. We thus sought to identify knowledge translation mechanisms, enabling factors and structures needed to effectively translate modelled evidence into policy decisions.

**Methods:**

We used convergent mixed methods in a participatory action approach, with quantitative data from a survey and qualitative data from a scoping review, in-depth interviews and workshop notes. Participants included researchers and policy actors involved in COVID-19 evidence generation and decision-making. They were mostly from lower- and middle-income countries (LMICs) in Africa, Southeast Asia and Latin America. Quantitative and qualitative data integration occurred during data analysis through triangulation and during reporting in a narrative synthesis.

**Results:**

We engaged 147 researchers and 57 policy actors from 28 countries. We found that the strategies required to use modelled evidence effectively include capacity building of modelling expertise and communication, improved data infrastructure, sustained funding and dedicated knowledge translation platforms. The common knowledge translation mechanisms used during the pandemic included policy briefs, face-to-face debriefings and dashboards. Some enabling factors for knowledge translation comprised solid relationships and open communication between researchers and policymakers, credibility of researchers, co-production of policy questions and embedding researchers in policymaking spaces. Barriers included competition among modellers, negative attitude of policymakers towards research, political influences and demand for quick outputs.

**Conclusion:**

We provide a contextualised understanding of knowledge translation for LMICs during the COVID-19 pandemic. Furthermore, we share key lessons on how knowledge translation from mathematical modelling complements the broader learning agenda related to pandemic preparedness and long-term investments in evidence-to-policy translation. Our findings led to the co-development of a knowledge translation framework useful in various settings to guide decision-making, especially for public health emergencies.

**WHAT IS ALREADY KNOWN ON THIS TOPIC**

- There has been a multitude of modelling frameworks used in diverse ways to advise the various pandemic responses the world over, to an extent not seen before in public health.
- However, it is likely that not all modelling and evidence was adequate, effectively communicated or used by policymakers.
- This is especially of concern in many lower- and middle-income countries (LMICs) that had strained health systems and resource constraints pre-pandemic.

**WHAT THIS STUDY ADDS**

- The know-do gap is a bottleneck to rapid, effective policy decisions, especially crucial in emergencies.
- As part of pandemic preparedness, it is necessary to have decision support systems in place.
- To ensure this is done well, there is a need to understand how modelling and analytical methods can rapidly be made available and fully integrated into decision-making processes.

**HOW THIS STUDY MIGHT AFFECT RESEARCH, PRACTICE OR POLICY**

- We identified mechanisms required to strengthen knowledge translation in LMICs, and this complements the broader learning agenda related to pandemic preparedness and long-term investments in evidence-to-policy translation.
- This study contributed to the co-development of a knowledge translation framework that will be useful in building model-to-policy systems that can be adapted for use in various settings.

## Introduction

 The COVID-19 pandemic is the most defining global health crisis of our time. According to the latest estimates, there have been over 700 million confirmed cases of COVID-19 and just over 7 million deaths globally.^1^ Besides directly causing death and disability, the pandemic also disrupted essential health services, putting additional stressors on health systems that were already under strain, especially in lower- and middle-income countries (LMICs).^2^,^5^ Policies to curb the spread of COVID-19 negatively affected economic growth and disrupted social services, leading to untold impacts—the pandemic disproportionately affected the most vulnerable.^2 4 6^

Understanding the magnitude of the effects of the pandemic on health and economic outcomes was essential to developing policies to respond to the crisis. At the pandemic’s beginning, policy decision-makers needed to know the fundamentals of the pathogen and the risk of spread. As it evolved, they needed to understand the incidence, hospitalisation and mortality rates, the effects of various pharmaceutical and non-pharmaceutical interventions and how to allocate resources optimally. As the pandemic subsided, the focus shifted to recovery and long-term impacts.^7^

Consequently, there was an unprecedented demand for modelling analytics to understand the pandemic and support various mitigation decisions. Compartmental models were commonly used during the pandemic to monitor individuals as they transition through various infection states (Susceptible-Infectious-Recovered (SIR) and Susceptible-Exposed-Infectious-Recovered models). Agent-based models were also widely used, employing computer simulations to generate a virtual environment where individuals follow defined rules.^8^ However, not all modelling and evidence were likely adequate, effectively communicated or effectively used by decision-makers.^9^,^11^

Despite considerable resources dedicated to research, transferring findings to practice is often a slow, unpredictable process and a bottleneck to rapid, evidence-based policy decisions needed in emergencies.^12^ This may have resulted in missed opportunities, wasted time and effort, and loss of life during the pandemic. It is, therefore, imperative to minimise the knowledge-to-action gap by understanding that knowledge translation processes occur in an environment of diverse evidence sources under uncertainty, with complex social interactions among various stakeholders. Dealing with uncertainties, mainly how to communicate them to decision-makers, is also a significant bottleneck.

**Figure 1:**
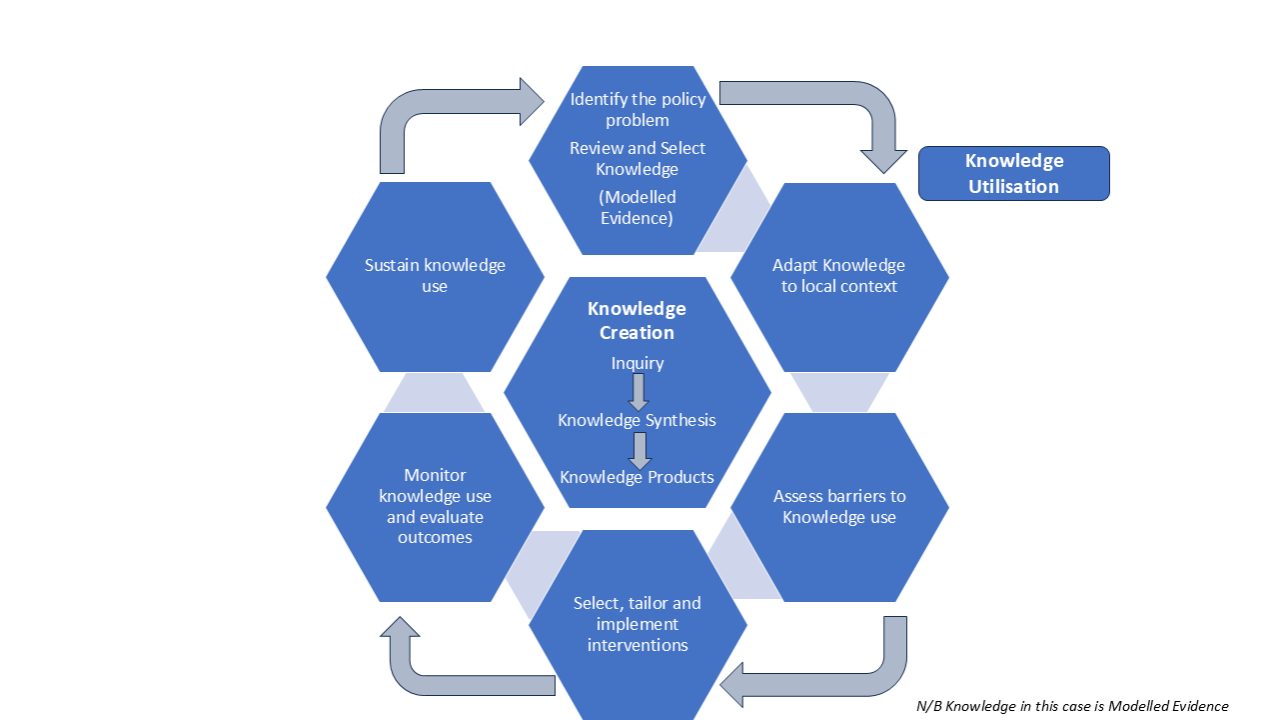
Creation and utilisation of knowledge from modelled evidence, based on Graham’s knowledge to action framework.^12^

Graham’s knowledge-to-action framework (illustrated in figure 1) has been tested as a model for planning and evaluating knowledge translation strategies.^12 13^ The framework is based on planned action theories. It divides the knowledge-to-action process into two concepts: (1) knowledge creation—where the researchers and policy actors generate policy-relevant questions and the relevant approaches to use them) and (2) utilisation—where the knowledge (modelled evidence in this case) is adapted to the local context and implemented. Guided by Graham’s framework, we set out to identify good practices, enabling factors, and structures needed to successfully create and use modelled evidence during the COVID-19 pandemic as a test case for future emergencies.

We found few studies that explicitly described knowledge translation strategies and how they were used to promote the uptake of modelled evidence for policy decision-making during the pandemic, and most of them were from higher-income settings.^14^,^16^ LMICs may have had limited modelling and knowledge translation capacity pre-pandemic, which may have hindered rapid decision-making during the pandemic.^17^ We therefore engaged both researchers and policy actors (primarily drawn from LMICs) to understand how modelling data was used for decision-making during the pandemic, what challenges they faced, and suggestions for improvement. This work resulted in the co-creation of a framework to guide evidence-based policy decision-making. It complements the broader learning agenda related to pandemic preparedness and investments in long-term improvement in evidence-to-policy translation.

## Methods

### Study design

We conducted a mixed methods study in a convergent manner, as described by Creswell and Plano Clark,^18^ using participatory action approaches^19^ to gain rich insights from triangulating the different data sources. Quantitative data were from an online survey, whose insights helped shape qualitative data collection from a scoping review, in-depth interviews and participant observer notes from learning workshops. Graham’s knowledge-to-action framework helped frame the study objectives, design the initial interview guides, and structure the analysis.

We carried out participatory action research through collective, self-reflective inquiry into the local context and social relationships, as described by Gilson *et al*.^20^ We did this through collaborative and introspective inquiry with participants by looking into their experiences around modelling, knowledge translation and evidence-based policy decision-making during the pandemic. The investigators were thus also participants in the workshops and participated in the co-creation process of the knowledge translation framework. As investigators, we tried to minimise bias by limiting our participation to mainly listening in and only stepping in to facilitate where needed. Our weekly data reflection meetings enabled us to exercise reflexivity to ensure the participants’ perceptions were captured.^21 22^ An authorship reflexivity statement is included in online supplemental file S1.

### Study setting and participants

We purposively selected participants mainly from Africa, Southeast Asia and Latin America to represent varied levels and capacities of knowledge translation of COVID-19 modelled evidence in lower-resourced settings. We included perspectives of higher-income participants who supported modelling efforts in the Global South. Various authors have defined successful knowledge translation as a demonstrable change in knowledge, skill or practice.^23 24^ We determined successful knowledge translation through collective sensemaking with participants on whether and how modelled evidence was considered or used during the public health decision-making process for the pandemic in their respective settings.

We did stakeholder mapping and a scoping review to identify key actors with interest and influence in the COVID-19 modelling knowledge translation space. This helped us generate a database of nearly 200 individuals from 28 countries. Stakeholders included researchers (epidemiologists, infectious disease modellers, economic modellers), with some from the Centres for Epidemiological Modelling and Analysis^25 26^ and The COVID-19 International Modelling Consortium—COMO)^27^; policy actors/decision makers (government officials, regional and global WHO representatives, task force/technical working group members); the COVID-19 Multi-Model Comparison Collaboration—CMCC^8^ with members from the World Bank, Health Intervention and Technology Assessment Programme-HITAP and WHO Head Quarters; intermediaries (knowledge brokers and boundary organisations); funders; non-governmental organisations; and the public through the media and patient support groups. (Online supplemental file S2 table 1 shows details of the stakeholders, the various countries they were from and the study activities they participated in.)

**Table 1:** Sociodemographic characteristics of study participants

Characteristic	Survey n (%) N=69	Interview n (%) N=25	Workshops n (%) N=110
Region			
Africa	30 (43%)	9 (36%)	60 (55%)
Asia	8 (12%)	8 (32%)	16 (15%)
Latin America	10 (15%)	7 (28%)	6 (5%)
Other	21 (30%)	1 (4%)	28 (25%)
Role played			
Researchers/modellers	47 (68%)	17 (68%)	83 (75%)
Policymakers	22 (32%)	8 (32%)	27 (25%)
Years in this role			
<5 years	20 (29%)	10 (40%)	
5–10 years	16 (23%)	12 (48%)	
11–19 years	19 (28%)	2 (8%)	
≥20 years	5 (7%)	1 (4%)	
Missing	9 (13%)	0	
Sex			
Male	32 (53%)	15 (60%)	
Female	27 (45%)	10 (40%)	
Other	1 (2%)	0	
Age group			
25–34 years	11 (18%)	3 (12%)	
35–44 years	23 (38%)	6 (24%)	
45–54 years	20 (33%)	14 (56%)	
55–64 years	6 (8%)	2 (8%)	
≥65 years	1 (2%)	0	
Missing	7 (10%)	0	

### Study procedures

**Figure 2:**
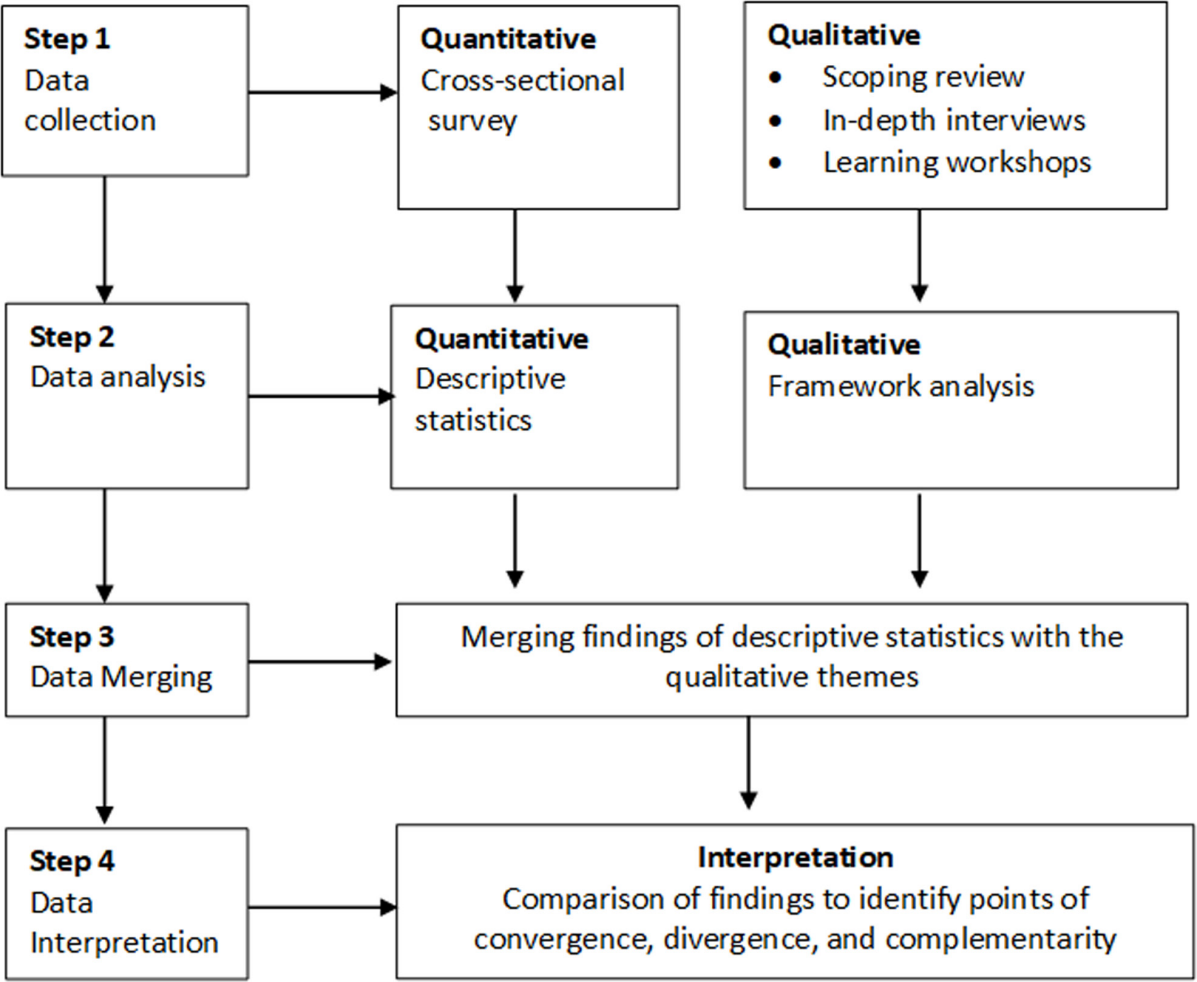
Description of the mixed methods approach.

The study involved a scoping review, an online survey, in-depth interviews and learning workshops as data sources. Figure 2 shows the convergent mixed method approach that we used.

We briefly detail the data collection and analysis processes in the subsequent section.

### Online survey

We distributed 156 survey questionnaires to potential stakeholders identified from our stakeholder mapping exercise and scoping review via email in May 2022. We translated and back-translated the surveys in French, Portuguese and Spanish and hosted them on QuestionPro^28^ (online supplemental file S3). We exported data from the survey into RedCAP software and ran consistency checks. We generated descriptive statistics on participant demographics, their knowledge creation and utilisation approaches and the enablers and structures needed for successful knowledge translation.

### Scoping review

We carried out a review to describe the knowledge strategies used to promote the uptake of COVID-19 modelled evidence by policy decision-makers. We searched the following databases: PubMed, Web of Science, MEDLINE, EMBASE, Arxiv, medRxiv, bioRxiv and grey literature for studies in English published between March 2020 and April 2022. The review was registered on OSF Registries.^29^ The details of the search strategy and included studies are in online supplemental file S4. FHG, JM and CJO used Rayyan and EndNote to screen titles and abstracts for eligibility. FHG and JM did full-text screening and data extraction. Any discrepancies were resolved through discussion with the study team.

### In-depth interviews and learning workshops

We identified key informants, mainly focusing on LMIC researchers and policy actors in two stages, initially purposively and then snowballing until the point of saturation. They shared their experiences translating modelled evidence to policy decisions during the pandemic. Graham’s framework initially informed semi-structured interview guides (online supplemental file S5) and was later adapted to explore survey and scoping review findings further. We conducted the interviews online between May and December 2022 due to COVID-19 restrictions.

Concurrently, we conducted three learning workshops with our stakeholders. The first online workshop was to get reflections from participants on their experiences with knowledge translation of modelled COVID-19 data. The second online workshop enabled sense-checking and initiated discussions on a knowledge translation guidance framework. The third hybrid workshop disseminated further study findings and continued with the co-development of the knowledge translation framework. The investigators were participants in these workshops and took observation notes.

We managed the interview transcripts, participant observation notes and scoping review using NVivo V.12 software. We conducted a thematic framework analysis^30^ to determine the knowledge creation and utilisation processes used during the pandemic, the participants’ perspectives of what approaches worked well, why and what could be done differently for future emergencies. CJO, JM and JJ read the transcripts and notes and generated initial codes. Discrepancies were discussed and refined during weekly team data reflection meetings. Similar codes were grouped into categories to form a working analytic framework. The process drew on the original research aims guided by Graham’s framework and analytical themes from the recurrence of views or experiences. Next came indexing and charting into a framework matrix. The final stage involved integrating qualitative and quantitative findings from the survey into a narrative synthesis. Anonymised quotes were used for illustrative purposes. Onlinesupplemental files S6  S7 have the codebook and snapshot of the framework analysis matrix, respectfully.

### Patient and public involvement

This study did not involve patients. Because of the participatory nature of our work, we involved the ‘public’ by recruiting policymakers and researchers who are consumers of our data throughout, from the design and conduct, and we have shared results with them.

## Results

We present the mechanisms used for knowledge translation of modelled evidence for policy decisions during the COVID-19 pandemic in lower-resourced settings, highlighting the enabling factors and infrastructural requirements for successful knowledge translation in future emergencies. GRAMMS criteria for reporting mixed methods studies guided us,^31^ and we illustrated key findings from the quantitative and qualitative arms and how we integrated them (online supplemental file S8).

We engaged 147 researchers and 57 policymakers/advisors from 20 LMICs and 8 high-income countries. Online supplemental file S2 tables 1–4 show the details of the countries from which the participants were drawn.

### Participants’ characteristics

Table 1 summarises the sociodemographic characteristics of study participants. The online survey had 69 respondents, 47 (68%) researchers and 22 (32%) policymakers; most (43%) were from Africa. Slightly more than half were male, and nearly 40% were aged 35–44. There were 25 interview respondents, 17 (68%) researchers and 8 (32%) policymakers, who were distributed across the three main regions (36% from Africa, 32% from Asia and 28% from Latin America). Most interviewees were male (60%), and more than half were aged 45–54. The three learning workshops had 110 participants, 83 (75%) researchers and 27 (25%) policymakers. The workshop attendees were mainly from Africa (55%), Asia (15%) and Latin America (5%).

### Findings from the stakeholder mapping exercise

**Figure 3:**
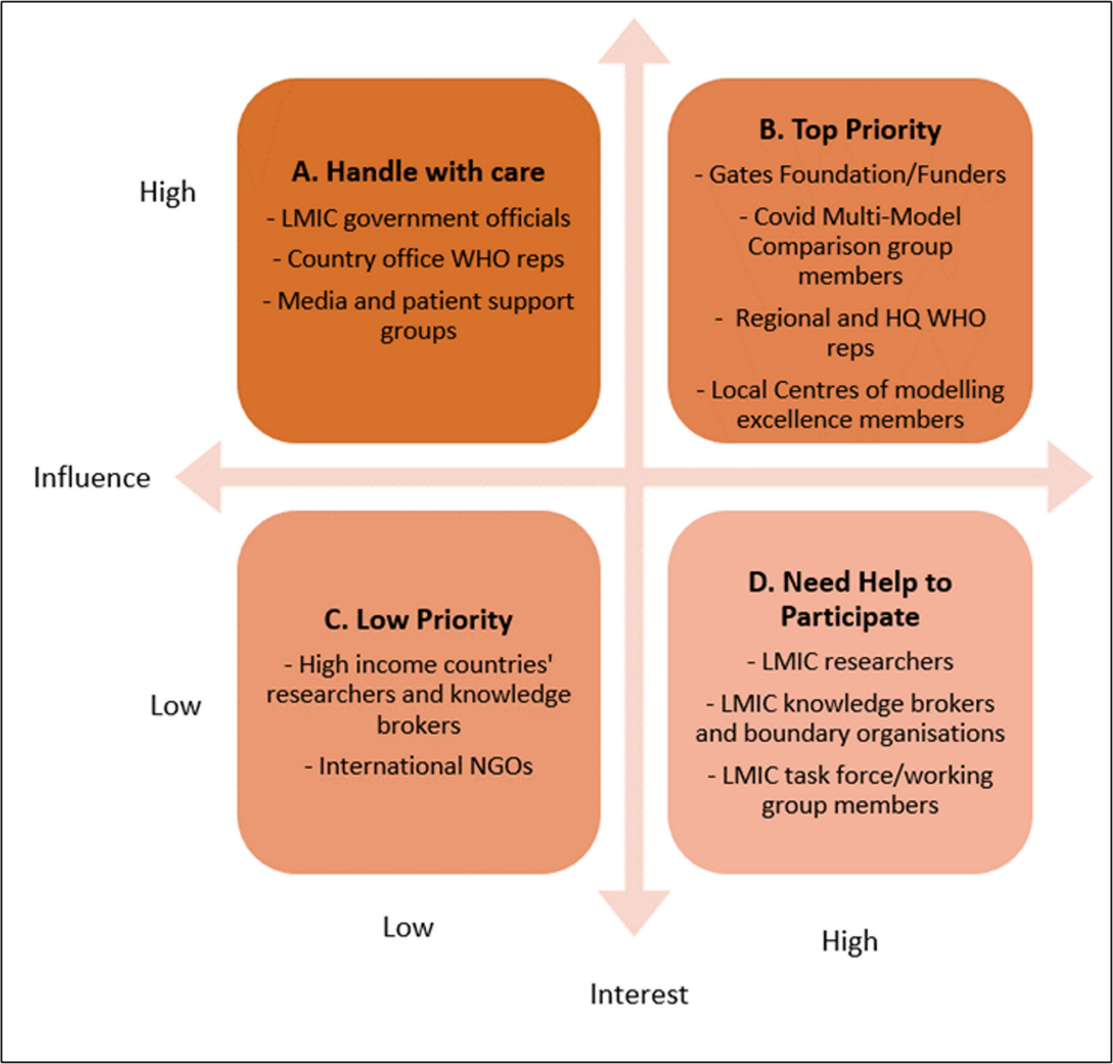
Stakeholder mapping. LMICs, lower- and middle- income countries; NGO, non-governmental organisation.

Figure 3 summarises findings from stakeholder mapping. The stakeholders in category A were highly influenced but had low interest and had to be handled with care/kept informed. They included local policy actors, country office representatives of WHO, local media and patient support groups. Category B was the high-influence and interest group, and we engaged actively with them throughout. They included representatives from members of centres of modelling excellence (the Centre for Epidemiological Modelling and Analysis (CEMA) in Kenya^25^ and the South African Centre for Epidemiological Modelling and Analysis (SACEMA)^26^ and the COVID-19 Modelling Consortium), the Gates Foundation, regional WHO offices and COVID-19 Modelling Comparison group members (with representation from the World Bank, WHO HQ and HITAP). Category C was the low-influence and low-interest stakeholders that we occasionally consulted mainly to get a developed world perspective, but they were not the focus of our study. This group comprised researchers and partner organisations from high-income settings. Finally, category D had high interest but low influence stakeholders, several of whom needed encouragement to participate. Several stayed engaged throughout. They included researchers, knowledge brokers/intermediaries and task force members from LMICs.

### Quantitative findings

#### Knowledge creation

**Figure 4:**
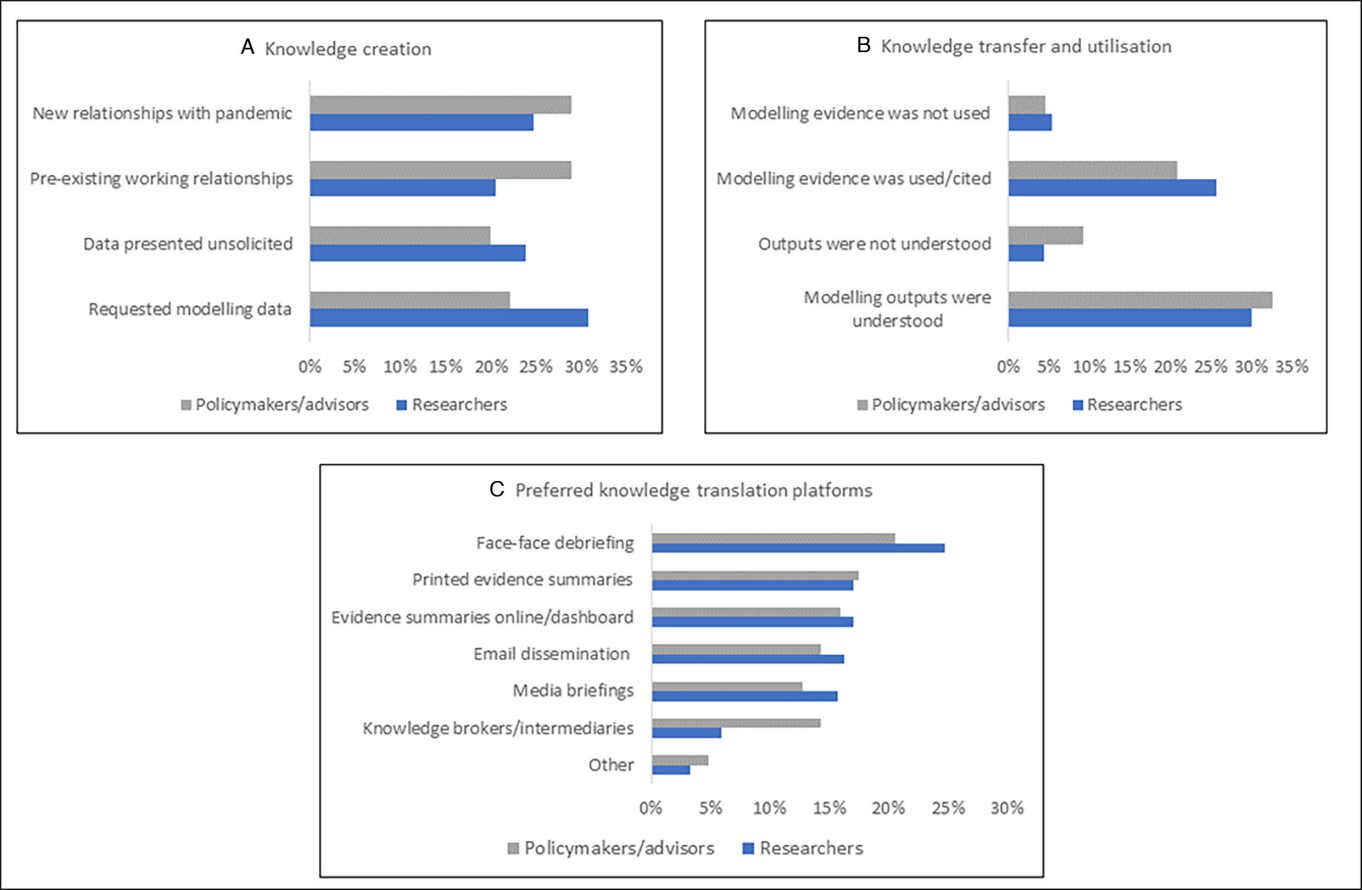
Panel with a summary of responses from survey participants.

We asked survey participants about the knowledge-creation approaches used during the pandemic, see panel figure 4A. Each proportion represents the number of survey participants from either researchers or policymakers’ groups who responded to each question. Approximately a third of the researchers commented that the policymakers requested modelling data from them, while around a quarter presented their data unsolicited. For the policymakers, almost a quarter reported requesting modelling data, whereas 20% received data unsolicited. 20% of researchers said they had working relationships with their counterpart policy actors before the pandemic. At the same time, around a quarter reported needing to develop new relationships to respond to the pandemic. Conversely, nearly 30% of policy actors reported having an existing relationship with researchers pre-pandemic, and an equal proportion reported needing to develop new relationships.

#### Knowledge transfer/utilisation

Participants’ responses to the knowledge transfer/utilisation queries are shown in figure 4B. Around a third of researchers felt that policy actors read and understood their modelling reports. Approximately a quarter responded that the modelling outputs they presented were used in policy decisions, and few perceived that their outputs were not used. A third of policymakers reported that they understood the modelled results presented to them, while nearly 10% acknowledged difficulties interpreting the data. A fifth said using model results to guide their decision-making; very few recognised that the data did not influence their policy decisions.

#### Preferred knowledge translation activities

When asked about knowledge translation activities, researchers and policymakers preferred face-to-face debriefing sessions, followed by printed policy briefs of evidence summaries on dashboards (figure 4C). Knowledge brokers/intermediaries were the least preferred platform by both researchers and policymakers.

### Qualitative findings

The scoping review summarised the knowledge translation strategies used to disseminate modelled evidence during the COVID-19 pandemic. Eight articles were eligible for full-text screening. They were from the WHO Eastern Mediterranean Region,^15^ the ECOWAS region,^16^ New Zealand,^32^ Hungary,^33^ Canada,^34^ Nigeria^14^ and two had Global representation.^27 35^ All the articles gave descriptive reports on how modelled evidence on COVID-19 was shared with policymakers. Other than information about who was targeted for the knowledge translation strategies, none of the studies provided details about the duration, frequency or timing of events or the personnel and resources required. The specific studies and critical lessons are summarised in online supplemental file S4 table 1.

In the subsequent section, we present emerging themes from the interviews and workshops and triangulate these with findings from the survey and scoping review in a narrative synthesis, highlighting enablers and structures required for effective knowledge translation.

### Enabling factors for knowledge translation

#### Working relationships between modellers and policymakers

Collaborative efforts between researchers (modellers) and policymakers before COVID-19 led to positive working relationships during the pandemic.

We’ve spent a lot of time developing relationships with the Ministry of Health and mechanisms for engaging with policymakers …essentially developing prior relationships with policymakers is important because it facilitated knowledge translation during the Covid-19 pandemic. (Researcher 04_Africa_Interview)

This corroborated survey findings showed that nearly a third of the researchers and policymakers reported having had pre-existing relationships.

In instances where relationships were poor, knowledge translation was ineffective because the policy actors were not open to advice. This frequently frustrated the researchers, as was observed in some settings.

But the … government didn’t take … any advice from us … The … government completely ignored the advice of … scientists … it was not easy dealing with the policymakers in most regions of X … The government was not very open to the suggestions … (Researcher 15_Latin America_Interview)

As the pandemic evolved, the need for urgent and timely research outputs occasionally led to strained relationships. Running models and packaging outputs required time, yet policymakers expected results quickly, which pressured modellers who worked around the clock to meet the demands.

… there’s always some sort of a clash regarding time and expectations between policymakers and researchers. But then how you address those working together over time so that you build those relationships? (Researcher 2_Africa_Interview)

In addition, researchers reported the need for some autonomy when building models, even as they acknowledged the need for good relationships and long-term engagement with policymakers before any crisis.

#### Communication

We noted that effective and regular communication between the researchers and the policymakers was fundamental to maintaining relationships. This was substantiated in the scoping review, where open communication was similarly noted as an enabling factor. Good communication ensured that urgent policy questions and research/modelling outputs were promptly exchanged and explained.

… it helped a lot to have very regular communication with government … it was really a back and forth, we would come forward with results to present them, and they would ask us for results as well … effective communication with government … was key to … make understanding and the uncertainty a little bit easier … (Researcher 5_Africa_Interview)

Where communication was ineffective, researchers sometimes sought alternative channels such as the media, which sometimes forced policy actors to consider advice from researchers due to public pressure.

… groups were engaging with the media out of frustration, because … they were not able to get through to the policymaking partners … (Researcher 7_Asia_Interview)

The media, therefore, became an essential channel for information exchange during the pandemic.

… we had a lot of work with the press, so we put a lot of effort into giving as many interviews as we can to ask, to reply to the questions that people were having, and also to use the press to spread the recommendations … (Researcher 15_Latin America_Interview)

#### Trust and credibility

Continuous engagement and communication created trust between researchers and policymakers, facilitating the uptake of research/modelling outputs if a researcher/institution was perceived to be credible.

I think it was also a thing of trust. It was this they knew they could rely on us … because of that relationship, cementing and this constant communication … people know us, and they come to us now … Right now, we get asked for things instead of even one going forward … (Researcher 5_Africa_Interview)

This was corroborated by workshop participants, one of whom emphasised that building trust takes time.

… building trust takes time. It can’t happen overnight during an emergency … (Researcher_Workshop 1)

Trust and credibility were key enablers of knowledge translation, as identified in the scoping review. Likewise, in the survey, most researchers reported that policymakers specifically requested COVID-19 modelling data from them to guide the decision-making, implying trust in their evidence.

#### Co-creation

Where trust and long-standing relationships existed, co-creation of evidence was possible. Co-creation here refers to the engagement of policymakers with researchers in knowledge generation processes, including generation and prioritisation of policy research questions, evidence synthesis, development of models and interpretation of model outputs. The co-creation process was perceived as a vital enabler in using modelled evidence to inform decisions on COVID-19 response strategies.

One of the strategies is coproduction of knowledge … a lot of the evidence generation processes we were … actively involving the policymakers … so that they become part of providing the solution … (Researcher and Policy Advisor_Africa_Interview)

We also noted this in the workshops, where participants reiterated the importance of co-creation in getting modelled evidence used in policy decision-making.

Other organizational-level factors that were important for COVID-19 were organisations that had documented processes for co-creating models or engaging the government from the beginning and model creation. And were much more successful at making sure that their models were … relevant to government priorities and policy needs … (Researcher, Workshop 1)

Furthermore, research institutions with previous collaborations in the knowledge creation process had established positive working relationships, which made their evidence more likely to be considered during the pandemic decision-making.

Institution Y has invested in co-creating research with the Ministry of Health, so all the COVID-19 research that we did over the past two years was a collaboration between Institution Y and the Ministry of Health … all our outputs have both Institution Y’s scientists and Ministry of Health employees. …. and we’ve been invited severally to make presentations to policymakers. (Researcher 4_Africa_Interview)

In the scoping view, policymakers’ involvement in the generation of evidence encouraged ownership of the process, which enabled them to use the evidence in decision-making. Similarly, in the survey, most policymakers reported working jointly with researchers to develop policy questions for decision-making during the pandemic.

#### Embeddedness

Besides, co-creation, an embedded approach where researchers were situated within policymaking spaces, was an effective way for researchers and policymakers to generate evidence collectively. We also identified some policymakers attached to research organisations to participate in knowledge generation. The participation of policymakers allowed them to have a better understanding of knowledge generation processes. Thus, they were better positioned to interpret and potentially use model outputs for decision-making.

… an embedded approach … we adopted a way of working with policymakers … we do that research within policymaking spaces and with active involvement and participation of policymakers … that has evolved over time and because of that it has really helped to facilitate the actual application of the research that we do. (Researcher 2_Africa_Interview)

Sometimes, researchers were invited to present their findings during live policy discussions. This was perceived to encourage the uptake of recommendations and their implementation.

… so that allows the scientists to come and attend those cabinet sessions if at all there is an issue on the agenda … is one of the greatest ways to have this evidence to the policymakers … (Policymaker 3_Africa_Interview)

### Structures needed for successful knowledge translation

#### Capacity building

We identified capacity building for local modelling expertise as critical for successful knowledge translation. Researchers and policymakers both underscored the importance of having several modellers who could generate context-relevant models for policy decision-making.

We have shortage of modelling expertise … policymakers wanted to have geographic specific interventions for Covid-19 response … we as a country, kept waiting for [researchers]to be able to guide but also inform us of which model and what is likely to work out where. (Policymaker 03, Africa_Interview)

Workshop participants suggested that having regional centres of excellence responsible for training modellers, fundraising, building collaborations and forming stable links with policymakers for co-creation purposes would be a good aspirational goal.

Beyond strengthening technical modelling capacity, the participants identified a need to train researchers and policymakers in science communication, specifically in disseminating scientific outputs to a lay audience. Several researchers found themselves in the deep end during the pandemic, having to communicate their findings with no prior training.

… it was hard, but we really didn’t have any previous training on how to do that … we had this big group, and we saw which were the good speakers, the people that had more clarity to spread the ideas, and then we just pushed them to do the interviews … (Researcher 15_Latin America_Interview)

Workshop participants also emphasised that policymakers needed training to empower them to interpret and use scientific evidence for decision-making. This included understanding the uncertainties in modelling and which questions can or cannot be answered by modelling data.

#### Data infrastructure

In addition to capacity building, the participants reported the need for local data systems and policies that made high-quality data available and enabled data sharing. Such infrastructure would allow modellers to develop timely models based on local data and with the capacity to inform local decision-making.

… important factors included the availability of high-quality local data and information systems that modellers could quickly pull up and use to develop models. It was helpful when these data systems were transparent and … formatted in a way that made them accessible and usable for modellers. (Researcher, Workshop 2)

The need for collaboration between academic institutions and the government to attain better data infrastructure was underscored, especially in low-resource settings.

… better data systems … improved surveillance, more open data, accessible data and then the other thing is more opportunities for collaboration between academia and government because you have to build trust. (Researcher 14_Latin America_Interview)

### Dedicated funding streams

Resources are needed to build capacity for technical modelling, knowledge translation expertise and data infrastructure. Respondents mentioned political buy-in as critical for governments to invest domestic public resources in modelling and for policymakers to use model outputs for decision-making. Therefore, researchers must build alliances within various levels of government that may lead to dedicated resource allocation for modelling.

… getting that political buy-in and the government to invest dedicated resources for modelling to respond to pandemics. I think it is a big challenge in low- and middle-income countries. If you look at countries in the West, most countries have a dedicated resource, a dedicated unit, or a dedicated university who have been assigned the responsibility and also the dedicated resources for those groups. (Researcher 11_Southeast Asia_Interview)

## Discussion

Our study helped us better understand the preferred mechanisms used for knowledge translation of modelled data during the COVID-19 pandemic, enabling factors and infrastructural requirements in preparation for future global emergencies, especially in LMIC settings

The commonly used knowledge translation activities were face-to-face debriefing, policy briefs and evidence summaries on dashboards. Relatedly, in studies from humanitarian emergencies, policymakers preferred research findings presented in short messages with key actions highlighted in bullet points or infographics on websites they could easily access and use.^36^ A study in the Mediterranean region likewise described the benefits of sharing research findings via non-technical audio-visual presentations and policy briefs.^15^ Surprisingly, we found that using knowledge brokers/intermediaries was the least popular knowledge translation mechanism; policymakers in LMICs preferred direct engagement with researchers. This is contrary to what was observed from studies from higher-income settings where knowledge brokers promoted a culture of using evidence for decision-making and thought to be an optimal knowledge translation and exchange strategy.^23 37^

As we explored enablers for effective knowledge translation of modelled evidence during the pandemic, pre-existing relationships between the researchers and policymakers, coupled with open communication, trust and credibility, were vital to co-creating policy-relevant knowledge products. In examples from Israel, Switzerland, Germany, Canada and South Korea, countries where the government collaborated well with scientists, seemed to have better patient outcomes during the pandemic compared with Italy, Spain, Brazil and the USA, where the government and scientists had more strained relationships with devastating consequences.^3338^,^41^ An example from LMICs, Nigeria, had a co-production approach through a presidential taskforce of decision-makers and multi-disciplinary academics. This enabled swift production and effective utilisation of scientific data in response to the COVID-19 pandemic.^14^ Kenya and South Africa similarly had participatory approaches of co-production and embeddedness, where researchers worked alongside policymakers to support collaborative research and learning processes pre-pandemic, which proved helpful in the pandemic responses.^20^

Barriers to effective knowledge translation included competition among modellers, negative attitude of policymakers towards research, political influences and demand for timeliness of research outputs for policy decisions. Policymakers sometimes cited difficulties in determining the quality of evidence, which has been reported in other studies.^42^ Some said that close integration caused a loss of autonomy and the impact of power dynamics on model quality.^14^ Findings from a review cited lack of time, limited access to research evidence, limited capacity to appraise and translate research evidence and resistance to change as some of the barriers to evidence-informed decision-making.^23^

Finally, we used participatory approaches to identify and prioritise structures required to navigate the barriers and support the effective translation of modelled evidence to policy, especially in lower-resourced settings. We packaged these in a framework and roadmap to guide policymakers that are described in detail elsewhere.^43^ A need for capacity building particularly stood out. Lower-resourced countries relied on collaborative efforts to cope during the pandemic. This was seen in action through efforts of groups like COMO^27^ and the emergence of centres of excellence like CEMA in Kenya^25^ and SACEMA.^26^ The pandemic also saw work from CMCC that provided guidance to ensure models were relevant, robust and useful for policy decision-making.^8 35^

### Framework to guide the use of modelling data to guide policy decision-making

**Figure 5:**
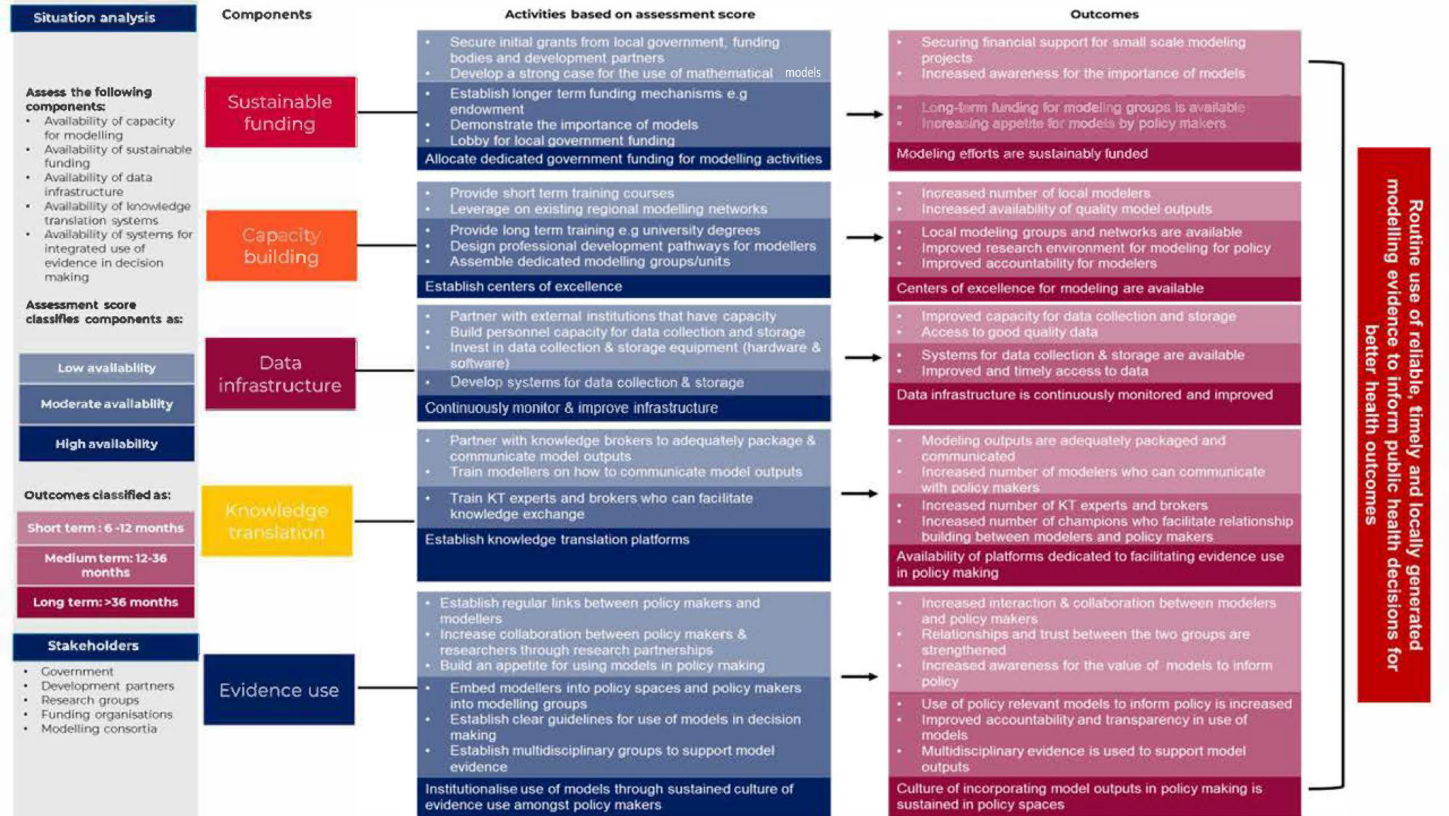
Framework to guide the use of models for policy decision-making.

With our findings and contributions from modellers and policymakers, we co-created a framework and implementation matrix to support using models for policy decision-making (figure 5), whose details are described further in this policy brief.^43^ The overall goal of the framework is to ensure the routine use of reliable, timely and locally generated modelled evidence to inform public health decisions for better population health outcomes. The five key components include (1) sustainable funding to support modelling for policy; (2) capacity building for modelling and knowledge transfer leading to centres of excellence; (3) sustainable systems and structures for the generation and use of model outputs; (4) dedicated platforms for knowledge translation; and (5) policymakers engaged throughout the knowledge generation process and thus committed to a culture of using evidence to guide decision making.

### Strengths and limitations

We used mixed methods, including an online survey, a scoping review, interviews and learning workshops, which gave us rich insights into knowledge translation mechanisms, what worked well, where and why, and how best to improve. Furthermore, the study findings contributed to the co-development of a knowledge translation roadmap and framework with an implementation matrix already well-received by policymakers in several participating countries.

The main limitation is that most of our study happened during the pandemic, restricting interviews and workshops to online platforms. This led to challenges such as interruptions due to unsteady internet connections, dealing with diverse time zones, difficulty building rapport and participation, and loss of non-verbal cues. We mitigated these by thorough preparation, offering data bundles to participants, scheduling times suitable for everyone, informal conversations before the formal sessions and skilful moderation to encourage participation. Despite the challenges, the online platform allowed us to eliminate geographical and time barriers and reach diverse participants worldwide, giving us rich data. When travel restrictions were lifted, we had one in-person/hybrid workshop that enabled the bulk of the co-creation component of our study due to deeper interactions.

## Conclusion

We provide a contextualised understanding of knowledge translation for LMICs during the COVID-19 pandemic. Face-to-face debriefings were the most preferred knowledge translation interventions. The critical enabling factor was pre-existing relationships between researchers and decision-makers. In addition, co-creation and embeddedness contributed to successful knowledge translation. Challenges identified included competition among modellers, the negative attitude of some policymakers towards science and political influence. Furthermore, we provide vital lessons on how knowledge translation from mathematical modelling complements the broader learning agenda related to pandemic preparedness and long-term investments in evidence-to-policy translation. The findings from this study led to the co-development of a knowledge translation framework that will be useful in integrating model-policy translation dynamics. The framework can be adapted in various settings to guide decision-making in preparation for and response to public health emergencies.

## Supplementary material

10.1136/bmjgh-2024-015247 online supplemental file 1

10.1136/bmjgh-2024-015247 online supplemental file 2

10.1136/bmjgh-2024-015247 online supplemental file 3

10.1136/bmjgh-2024-015247 online supplemental file 4

10.1136/bmjgh-2024-015247 online supplemental file 5

10.1136/bmjgh-2024-015247 online supplemental file 6

10.1136/bmjgh-2024-015247 online supplemental file 7

10.1136/bmjgh-2024-015247 online supplemental file 8

## Data Availability

Data are available upon reasonable request.

## References

[1] World Health Organisation (2023). WHO coronavirus (covid-19) dashboard. https://covid19.who.int/.

[2] Haleem A, Javaid M, Vaishya R (2020). Effects of COVID-19 pandemic in daily life. *Curr Med Res Pract*.

[3] The United Nations Department of Economic and Social Affairs (UN DESA) Everyone included: social impact of COVID-19. https://www.un.org/development/desa/dspd/everyone-included-covid-19.html.

[4] Pak A, Adegboye OA, Adekunle AI (2020). Economic Consequences of the COVID-19 Outbreak: the Need for Epidemic Preparedness. Front Public Health.

[5] Veettil SK, Schwerer L, Kategeaw W (2023). Scoping review of modelling studies assessing the impact of disruptions to essential health services during COVID-19. BMJ Open.

[6] World Health Organisation (2020). Impact of covid-19 on people’s livelihoods, their health and our food systems. https://www.who.int/news/item/13-10-2020-impact-of-covid-19-on-people’s-livelihoods-their-health-and-our-food-systems.

[7] Adiga A, Dubhashi D, Lewis B (2020). Mathematical Models for COVID-19 Pandemic: A Comparative Analysis. *J Indian Inst Sci*.

[8] Clapham H, Gad M, Gheorghe A (2021). Assessing fitness-for-purpose and comparing the suitability of COVID-19 multi-country models for local contexts and users. Gates Open Res.

[9] Cairney P, Wellstead A (2021). COVID-19: effective policymaking depends on trust in experts, politicians, and the public. Pol Des Pract.

[10] Colman E, Wanat M, Goossens H (2021). Following the science? Views from scientists on government advisory boards during the COVID-19 pandemic: a qualitative interview study in five European countries. BMJ Glob Health.

[11] Nixon K, Jindal S, Parker F (2022). Real-time COVID-19 forecasting: challenges and opportunities of model performance and translation. Lancet Dig Health.

[12] Graham ID, Logan J, Harrison MB (2006). Lost in knowledge translation: Time for a map?. J Contin Educ Health Prof.

[13] Ward V, House A, Hamer S (2009). Developing a Framework for Transferring Knowledge Into Action: A Thematic Analysis of the Literature. *J Health Serv Res Policy*.

[14] Abubakar I, Dalglish SL, Ihekweazu CA (2021). Lessons from co-production of evidence and policy in Nigeria’s COVID-19 response. BMJ Glob Health.

[15] Adib K, Hancock PA, Rahimli A (2021). A participatory modelling approach for investigating the spread of COVID-19 in countries of the Eastern Mediterranean Region to support public health decision-making. BMJ Glob Health.

[16] Sombié I, Johnson E, Lokossou V (2020). How does the West African Health Organisation (WAHO) contribute to the evidence based decision-making and practice during COVID-19 pandemic in ECOWAS region?. Pan Afr Med J.

[17] Silal S, Bardsley C, Menon R (2022). Epidemiological Modelling for Public Health Decision-Making in Sub-Saharan Africa: A Strategic Plan for Capacity Strengthening.

[18] Creswell JW, Plano Clark VL (2011). Designing and Conducting Mixed Methods Research.

[19] Baum F, MacDougall C, Smith D (2006). Participatory action research. J Epidemiol Community Health.

[20] Gilson L, Barasa E, Brady L (2021). Collective sensemaking for action: researchers and decision makers working collaboratively to strengthen health systems. BMJ.

[21] Bergen N, Labonté R (2020). “Everything Is Perfect, and We Have No Problems”: Detecting and Limiting Social Desirability Bias in Qualitative Research. Qual Health Res.

[22] Molyneux S, Sukhtankar P, Thitiri J (2021). Model for developing context-sensitive responses to vulnerability in research: managing ethical dilemmas faced by frontline research staff in Kenya. BMJ Glob Health.

[23] Dobbins M, Hanna SE, Ciliska D (2009). A randomized controlled trial evaluating the impact of knowledge translation and exchange strategies. Implement Sci.

[24] LaRocca R, Yost J, Dobbins M (2012). The effectiveness of knowledge translation strategies used in public health: a systematic review. BMC Public Health.

[25] Centre for Epidemiological Modelling and Analysis (CEMA) (2023). A center of excellence using data-driven approaches to control infectious diseases in Kenya & Africa university of Nairobi. https://cema.africa/.

[26] South African Centre for Epidemiological Modelling and Analysis (SACEMA) (2023). Modelling and analysis to improve health in Africa Stellenbosch. https://www.sacema.org/.

[27] Aguas R, White L, Hupert N (2020). Modelling the COVID-19 pandemic in context: an international participatory approach. BMJ Glob Health.

[28] QuestionPro https://www.questionpro.com/.

[29] Guleid F, Oliwa J, Maluni J (2022). Uptake of covid-19 modelling evidence to inform policy making using knowledge translation: a scoping review protocol. https://osf.io/65kcg.

[30] Srivastava A, Thomson SB (2009). Framework analysis: A qualitative methodology for applied policy research. J Admin Gov.

[31] O’Cathain A, Murphy E, Nicholl J (2008). The quality of mixed methods studies in health services research. J Health Serv Res Policy.

[32] Hendy S, Steyn N, James A (2021). Mathematical modelling to inform New Zealand’s COVID-19 response. J R S N Z.

[33] Gombos K, Herczeg R, Erőss B (2021). Translating Scientific Knowledge to Government Decision Makers Has Crucial Importance in the Management of the COVID-19 Pandemic. Popul Health Manag.

[34] Hillmer MP, Feng P, McLaughlin JR (2021). Ontario’s COVID-19 Modelling Consensus Table: mobilizing scientific expertise to support pandemic response. Can J Public Health.

[35] Teerawattananon Y, Kc S, Chi Y-L (2022). Recalibrating the notion of modelling for policymaking during pandemics. Epidemics.

[36] Khalid AF, Grimshaw JM, Parakh ND (2023). Decision-makers’ experiences with rapid evidence summaries to support real-time evidence informed decision-making in crises: a mixed methods study. BMC Health Serv Res.

[37] van Kammen J, de Savigny D, Sewankambo N (2006). Using knowledge brokering to promote evidence-based policy-making: The need for support structures. Bull World Health Organ.

[38] Moeckli B, Peloso A, Oldani G (2020). The Swiss approach to the COVID-19 outbreak. Am J Transplant.

[39] Oh J, Lee J-K, Schwarz D (2020). National Response to COVID-19 in the Republic of Korea and Lessons Learned for Other Countries. *Health Syst Reform*.

[40] Yamey G, Gonsalves G (2020). Donald Trump: a political determinant of covid-19. BMJ.

[41] de Almeida L, Carelli PV, Cavalcanti NG (2022). Quantifying political influence on COVID-19 fatality in Brazil. PLoS One.

[42] Muscatello DJ, Chughtai AA, Heywood A (2017). Translation of Real-Time Infectious Disease Modeling into Routine Public Health Practice. *Emerg Infect Dis*.

[43] Guleid F, Oliwa J (2023). Policy brief: framework to guide the use of mathematical modelling in evidence-based policy decision making. KEMRI-Wellcome Trust Research Programme.

