# Supplementary material for: Lessons learned from COVID-19 modelling efforts for policy decision-making in lower- and middle-income countries
Source: BMJ Glob Health. 2024 Nov 8;9(11):e015247. doi: 10.1136/bmjgh-2024-015247 (PMC11552008; doi:10.1136/bmjgh-2024-015247)
Supplement: online supplemental file 1 [file bmjgh-9-11-s001.pdf]

Supplementary File S1Author reflexivity Statement

Study conceptualisation

How does this study address local research and policy priorities?

We identified that modelling and use of evidence to guide policy decision-making had been a gap in many lower-middle-income countries (LMICs), which became more apparent during the COVID-19 pandemic, where rapid decision-making with far-reaching social and economic impacts was needed. Our work provided a contextualised understanding of knowledge translation for LMICs during the COVID-19 pandemic, sharing vital lessons on how knowledge translation from mathematical modelling complements the broader learning agenda related to pandemic preparedness and long-term investments in evidence-to-policy translation.

How were local researchers involved in study design?

CJO, FG, JKM, JJ, VW, JN and JO are LMIC researchers and co-investigators in this study through a Gates Foundation grant awarded to JO. They were all involved in conceptualisation of the study, in data collection, analysis and interpretation of findings.

Research management

How has funding been used to support the local research team(s)?

Funding from the Gates Foundation enabled a postdoc fellowship for JO, and FG developed her PhD concept building from this study.

CJO and JKM did short courses on knowledge translation.

Data acquisition and analysis

How are research staff who conducted data collection acknowledged?

They are all co-authors in this manuscript

How have research partnership members been provided access to study data?

They took part in data collection and analysis and had access to the data.

How were data used to develop analytical skills within the partnership?

Junior team members (CJO, FG, JKM, and JJ) received support and mentorship in knowledge translation, participatory action research, stakeholder engagement, and analysis of mixed methods research.

Data interpretation

How have research partners collaborated in interpreting study data?

Data analysis was done through weekly data reflection meetings where team members reviewed the data as it was collected and reflected on emerging themes.

Drafting and revising for intellectual content

How were research partners supported to develop writing skills?

All of the authors had prior experience in writing peer-reviewed manuscripts.

How will research products be shared to address local needs?

We prepared a policy brief that has been circulated to stakeholders of all the participating countries and we have two manuscripts from the work that we hope to be published in open-access journals.

Authorship

How is the leadership, contribution and ownership of this work by LMIC researchers recognised within the authorship?

CJO, FG, JKM, JM, VW, JN, and JO are all LMIC authors.

CJO first author and JO senior author.

How have early career researchers across the partnership been included within the authorship team?

CJP, FG, JKM, and JJ are all early career researchers, and the senior author, JO, is an early career postdoc researcher who won an award to do this work.

How has gender balance been addressed within the authorship?

FG, JKM, JJ, SYS, BLH, JN, JO- seven authors are female

CJO, VW, RH- three authors are male

Training

How has the project contributed to training of LMIC researchers?

Junior team members (CJO, FG, JKM, and JJ) received support and mentorship in knowledge translation, participatory action research, stakeholder engagement, and analysis of mixed methods research.

CJO and JKM took a short course in knowledge translation

Infrastructure

How has the project contributed to improvements in local infrastructure?

The grant was not geared towards infrastructure development.

Governance

What safeguarding procedures were used to protect local study participants and researchers?

The study was approved by The KEMRI (Kenya Medical Research Institute) Scientific and Ethics Review Unit (SERU) after thoroughly reviewing the protocol to ensure that procedures were ethically sound, and participants were protected. We ensured the anonymity of all our

respondents and kept data safe in password-protected project computers only accessible to research staff in the project.
