# Supplementary material for: Lessons learned from COVID-19 modelling efforts for policy decision-making in lower- and middle-income countries
Source: BMJ Glob Health. 2024 Nov 8;9(11):e015247. doi: 10.1136/bmjgh-2024-015247 (PMC11552008; doi:10.1136/bmjgh-2024-015247)
Supplement: online supplemental file 2 [file bmjgh-9-11-s002.pdf]

Supplemental File S2 Study Participants

Table 1 Countries that participated in the study

Country	Region	Country Classification (World Bank)	Survey	Interviews	Learning Workshops
1. Kenya	Africa	LMIC	X	X	X
2. South Africa		UMIC	X	X	X
3. Ghana		LMIC	X		
4. Congo		LMIC	X		
5. Nigeria		LMIC	X		X
6. Rwanda		LIC	X		X
7. Uganda		LIC	X	X	X
8. Tunisia		LMIC		X	X
9. Thailand	Asia	UMIC	X	X	X
10. India		LMIC	X		
11. Malaysia		UMIC	X	X	X
12. Singapore		HIC	X	X	X
13. Kyrgyzstan		LMIC	X	X	
14. Argentina	Latin America	UMIC	X	X	
15. Brazil		UMIC	X	X	
16. Panama		HIC	X		
17. Colombia		UMIC	X		
18. Ecuador		UMIC	X		
19. Chile		HIC	X	X	
20. Paraguay		UMIC	X		
21. UK	Other regions	HIC	X		X
22. USA		HIC	X		X
23. Switzerland		HIC	X		X

24. Australia		HIC	X		X
25. France		HIC			X
26. Netherlands		HIC			X
27. New Zealand		HIC			X
28. Canada		HIC			X

Table 2 Survey Respondents

Region	Country	Researchers	Policymakers	Total
**Africa**	Kenya	11	8	**19**
	South Africa	2	2	**4**
	Multiple Countries	1	1	**2**
	Ghana	0	1	**1**
	Congo	0	1	**1**
	Nigeria	1	0	**1**
	Rwanda	1	0	**1**
	Uganda	1	0	**1**
**Southeast Asia**	Thailand	1	2	**3**
	India	0	1	**1**
	Malaysia	1	0	**1**
	Singapore	3	0	**3**
**Latin America**	Argentina	2	0	**2**
	Brazil	1	0	**1**
	Panama	1	0	**1**
	Colombia	2	1	**3**
	Ecuador	1	0	**1**
	Chile	1	0	**1**
	Paraguay	1	0	**1**

**Others**	UK	10	1	**11**
	Multiple	2	0	**2**
	USA	2	2	**4**
	Switzerland	0	2	**2**
	Kyrgyzstan	1	0	**1**
	Australia	1	0	**1**
**TOTAL**		**47**	**22**	**69**

Table 3 Interviews Respondents

**Region**	**Country**	**Researchers**	**Policymakers**	**Total**
**Africa**	Kenya	4	1	**5**
	South Africa	1	0	**1**
	Multiple Countries	0	1	**1**
	Uganda	0	1	**1**
	Tunisia	1	0	**1**
**Southeast Asia**	Thailand	4	1	**4**
	Malaysia	2	0	**2**
	Singapore	1	0	**1**
**Latin America**	Argentina	0	1	**1**
	Brazil	2	3	**5**
	Chile	1	0	**1**
**Others**	Kyrgyzstan	1	0	**1**
**TOTAL**		**17**	**8**	**25**

Table 4 Learning workshop participants

Region	Country	Researchers	Policymakers	Total
**Africa**	Kenya	38	6	**44**
	South Africa	2	1	**3**
	Multiple countries	0	3	**3**
	Uganda	0	2	**2**
	Tunisia	3	0	**3**
	Nigeria	3	0	**3**
	Rwanda	2	0	**2**
**Southeast Asia**	Thailand	7	4	**11**
	Malaysia	3	0	3
	Singapore	2	0	**2**
**Latin America**	Ecuador	1	0	**1**
	Argentina	0	2	**2**
	Brazil	2	0	**2**
	Chile	1	0	**1**
**Others**	Switzerland	0	4	**4**
	France	1	0	**1**
	UK	10	0	**10**
	USA	6	2	**8**
	Australia	0	1	**1**
	Netherlands	1	0	**1**
	New Zealand	0	2	**2**
	Canada	1	0	**1**
**TOTAL**		**83**	**27**	**110**
