# Supplementary material for: Lessons learned from COVID-19 modelling efforts for policy decision-making in lower- and middle-income countries
Source: BMJ Glob Health. 2024 Nov 8;9(11):e015247. doi: 10.1136/bmjgh-2024-015247 (PMC11552008; doi:10.1136/bmjgh-2024-015247)
Supplement: online supplemental file 3 [file bmjgh-9-11-s003.pdf]

# Supplementary File S3 Online Survey tool

This section is to collect general information about you and the professional role (s) that you play.

1. Indicate your gender

☐ Male
☐ Female
☐ Other

2. Indicate which age group you fall under

☐ <25yr
☐ 25-34yr
☐ 35-44yr
☐ 45-54yr
☐ 55-64yr
☐ ≥65yr

3. Indicate which region you work (Specify Country)

☐ Africa ________________________________________
☐ South East Asia________________________________
☐ North America________________________________
☐ Central/South America_________________________
☐ Europe_______________________________________
☐ Eastern Mediterranean_________________________
☐ Western Pacific________________________________

4. Specify which professional role you play

☐ Researcher/modeller: (Specify the type of modelling area specific to COVID-19, e.g., economic modelling, epidemiological modelling, etc.)

☐ Policymaker

5. Specify time spent in this role

☐ <5yr
☐ 5-10 yr.
☐ 11-14yr
☐ 15-20yr
☐ >20yr

6. For researchers, any experience sharing your COVID-19 modelling data outputs with policy decision-makers:

☐ Yes
☐ No

7. For researchers, if yes, select all statements that reflect your perceptions of what is relevant about your experiences about sharing COVID-19 modelling data outputs with policymakers:

☐ The policymakers requested COVID-19 modelling data from you/your group to guide their decision-making during the pandemic

☐ You/your group presented COVID-19 modelling data to policymakers (unsolicited)

☐ You/your group have a pre-existing working relationship with policymakers and have been working together to create policy questions and generate evidence for decision-making before the pandemic

☐ You/your group developed a new working relationship with policymakers during the pandemic and are now working together to create policy questions and generate evidence for decision-making

☐ My/our group's COVID-19 modelling reports were read and understood by policymakers (you received positive feedback from them/the evidence you shared was referenced/cited by them)

☐ My/our group's COVID-19 modelling reports were not read and understood by policymakers (you have not received any positive feedback from them)

☐ Policymakers reported having difficulties understanding my/our COVID-19 modelling data reports

☐ I have had training in science communication/disseminating my findings to a lay audience

☐ My group has a science communicator/engages with science communicators to help communicate our findings to a lay audience

☐ My/our group's COVID-19 modelling reports were utilised as they were by policymakers (you received positive feedback from them/the evidence you shared was referenced/cited by them)

☐ My/our group's COVID-19 modelling reports were not utilised by policymakers/did not inform or influence policy decisions (you have not received positive feedback from them)

☐ My/our group's COVID-19 modelling reports influenced choices and decisions by policymakers (you received positive feedback from them about adapting your findings)

☐ My/our group's COVID-19 modelling reports did not influence the choices and decisions of policymakers (you have not received positive feedback from them)

☐ Other (specify)________________________________________

8. For researchers, tick all the relevant methods you/your group used to share/disseminate your COVID-19 modelling data outputs with policymakers

☐ Printed policy briefs/brochures of evidence summaries in lay language

☐ Face-to-face debriefing sessions

☐ Evidence summaries posted on the group’s website/dashboards/blogs

☐ Email dissemination of evidence summaries

☐ Use an intermediary/knowledge broker/science communicator to simplify your findings and share them with policymakers

☐ Media briefings of evidence summaries in lay language

☐ Other (specify)________________________________________

9. For researchers (please respond whether you ticked yes or no to Q 6), give your suggestions of what you could have done differently/can be done to better engage with policymakers to disseminate your COVID-19 modelling outputs

______________________________________________________________________

_______________________________________________________________________________

10. For policymakers, any experience receiving COVID-19 modelling data outputs from researchers to aid your decision-making process

☐ Yes
☐ No

11. For policymakers, if yes, select all statements that reflect your perceptions of what is relevant about your experiences about receiving COVID-19 modelling data outputs from researchers:

☐ I/we requested COVID-19 modelling data from researchers/modellers to guide the decision-making during the pandemic

☐ I/we had researchers present their COVID-19 modelling data to us (unsolicited)

☐ I/we have a pre-existing working relationship with researchers and have been working together to create policy questions and generate evidence for decision-making before the pandemic

☐ I/we have developed a new working relationship with researchers during the pandemic and are now working together to create policy questions and generate evidence for decision-making

☐ I read and understood information from COVID-19 modelling data reports presented to me

☐ I read but had difficulties understanding the COVID-19 modelling data reports presented to me

☐ I/we have utilised COVID-19 modelling reports as they were to guide decision-making

☐ I/we adapted COVID-19 modelling reports from settings similar to ours to guide decision-making

☐ COVID-19 modelling reports influenced my/our choices and decisions related to the pandemic (Specify which ones, e.g., epidemiological, scenario forecasts, economic models, etc.)

☐ COVID-19 modelling reports did not necessarily influence my/our choices and decisions related to the pandemic

Specify any other experience, positive or negative, that you had trying to utilise COVID-19 modelling reports for your decision-making.

_______________________________________________________________________________
_______________________________________________________________________________

12. For policymakers, tick all the relevant methods you prefer to receive COVID-19 modelling data outputs from researchers

☐ Printed policy briefs/brochures of evidence summaries in lay language

☐ Face-to-face debriefing sessions

☐ Evidence summaries posted on the group's website/dashboards/blogs

☐ Email dissemination of evidence summaries

☐ Use an intermediary/knowledge broker/science communicator to simplify your findings and share them with policymakers

☐ Media briefings of evidence summaries in lay language

☐ Other (specify)_____________________________________________

13. For policymakers, (please respond whether you ticked yes or no to Q 10) give your suggestions of what you could have done differently/can be done to better engage with researchers to support your decision-making efforts

_______________________________________________________________________________

Thank you very much for the time you have dedicated to this very important initiative. The findings of this study will help us understand how to improve the utilisation of COVID-19 modelling data for decision-making as we combine efforts to mitigate the disastrous effects of the ongoing pandemic. We will present results to key stakeholders in workshops, which you are invited to be a part of. We will also share written reports of our findings with you, including actionable recommendations from the lessons learned.
