# Supplementary material for: Lessons learned from COVID-19 modelling efforts for policy decision-making in lower- and middle-income countries
Source: BMJ Glob Health. 2024 Nov 8;9(11):e015247. doi: 10.1136/bmjgh-2024-015247 (PMC11552008; doi:10.1136/bmjgh-2024-015247)
Supplement: online supplemental file 4 [file bmjgh-9-11-s004.pdf]

Supplementary File S4 Scoping Review

This review aimed to describe and combine the different Knowledge Translation strategies used to share modelling evidence with policymakers during the COVID-19 pandemic. It identified the barriers and facilitators of the uptake of modelling evidence and shared learnings from these processes. It was registered on OSF here: https://osf.io/65kcg

Review question: What and how have knowledge translation (KT) approaches/strategies been used to promote uptake of COVID-19 modelling evidence for policy making?

Objective: To provide an overview and appraisal of published articles and grey literature that describe KT approaches used to promote uptake of COVID-19 modelling evidence for decision making.

Specific objectives:

- Describe KT strategies and/or tools used in translating modelling evidence during the pandemic
- Identifying barriers and facilitators to using KT strategies intended to promote uptake of COVID-19 modelling evidence for decision making
- Identify outcomes if reported for those KT approaches

Eligibility Criteria

Type of studies

All study designs that presented evidence from mathematical models of COVID-19 were considered for inclusion in the review. Additionally, we included articles from grey literature, including reports from government and research institution websites.

Participants

Only studies that involved local and international decision-makers making policy-level decisions on the COVID-19 pandemic were included.

Intervention

Studies that described the facilitation of using modelling evidence in COVID-19 decision-making were included in the review. The review excluded any KT interventions that did not support policy-level decision-making.

Search Strategy

The following search strategy was used:

Table: Search strings used in the scoping review. Concepts were combined using the Boolean operator "AND"

**Concept 1: Knowledge translation**	**Concept 2: COVID-19**	**Concept 3: Modelling**
knowledge OR evidence OR finding* OR research OR synthesis) AND ("research to action" OR "decision mak*" OR "policy mak*" OR policy OR decision OR "evidence-informed decision making" OR "evidence to policy" OR participat*)) AND (appl* or broke* or creation or diffus* or disseminat* or exchang* or implement* or manage* or mobili* or translat* or transfer* or uptak* or utili* OR "knowledge translation" OR kt	((("COVID-19"[Mesh] OR "SARS-CoV-2"[Mesh] OR "COVID-19 Vaccines"[Mesh] OR "COVID-19 Serological Testing"[Mesh] OR "COVID-19 Nucleic Acid Testing"[Mesh] OR "SARS-CoV-2 variants" [Supplementary Concept] OR "COVID-19 drug treatment" [Supplementary Concept] OR "COVID-19 serotherapy" [Supplementary Concept] OR "2019-nCoV" OR "2019nCoV" OR "cov 2" OR "Covid-19" OR "sars coronavirus 2" OR "sars cov 2" OR "SARS-CoV-2" OR "severe acute respiratory syndrome coronavirus 2" OR "coronavirus 2" OR "COVID 19" OR "COVID-19" OR "2019 ncov" OR "2019nCoV" OR "corona virus disease 2019" OR "cov2" OR "COVID-19" OR "COVID19" OR "nCov 2019" OR "nCoV" OR "new corona virus" OR "new coronaviruses" OR "novel corona virus" OR "novel coronaviruses" OR "SARS Coronavirus 2" OR "SARS2" OR "SARS-COV-2" OR "Severe Acute Respiratory Syndrome Coronavirus 2"))	("mathematical model*" OR model* OR predict* OR dynamic* OR estimat* OR forecast* OR "economic model*" OR likelihood

Identifying studies

The review attempted to include all relevant published and unpublished studies. Electronic searches were conducted in the following databases: PubMed, Web of Science, MEDLINE, EMBASE, Arxiv, medRxiv and bioRxiv and google for grey literature. We also searched the references of included studies for additional articles to include in the review. To identify grey literature, we searched websites

of policy and research institutions for reports and policy documents. Finally, we ran google and google scholar searches. Only studies in English that were published between March 2020 to April 2022 were included.

Data collection and analysis

Retrieved articles were collected and uploaded into Rayyan and EndNote and duplicates removed. Titles and abstracts were then screened by three independent reviewers (FHG, JM and CO) for assessment against the eligibility criteria for the review. Full text screening of the remaining studies against the inclusion criteria were conducted by two independent reviewers (FHG & JM). Any disagreements that arose between the reviewers at each stage of the selection process were resolved through discussion, or with an additional reviewer/s.

Data extraction/charting

Data was extracted independently by 2 reviewers (FHG & JM) using a data extraction tool developed by the reviewers. Extracted items included:

1. General characteristics of included papers (country of study, type of modelling, type of study etc.)

2. KT strategies used including target audience

3. Barriers and facilitators

4. Outcomes (if reported)

Throughout data extraction, the tool was adapted and revised as necessary.

Data analysis

A narrative synthesis was used to describe and summarise the extracted results and discuss how the results answer the review question/s.

Selection process

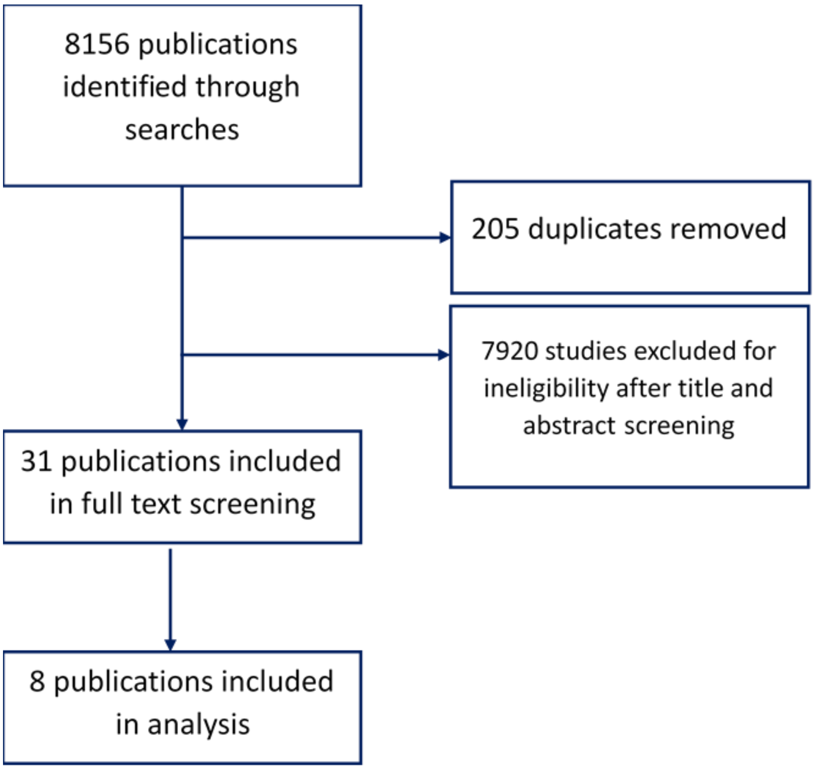

Figure 1 Selection process for the scoping review

Table Scoping review: Characteristics of included studies

Author	Study	Region	Experiences/ Findings
Adib et al.2021 [15]	A participatory modelling approach for investigating the spread of COVID-19 in countries of the Eastern Mediterranean Region to support public health decision-making	Mediterranean	• Limited surveillance data<br>• Designed a participatory process to develop rapid modelling analyses tailored to the COVID-19 epidemic situation.
Abubakar et al.2021 [14]	Lessons from co-production of evidence and policy in Nigeria's COVID-19 response	Africa - Nigeria	• Limited transparency<br>• Bureaucratic obstacles<br>• Focus on epidemiological approaches
Sombie et al, 2020 [16]	How does the West African Health Organisation (WAHO) contribute to the evidence-based decision making and practice during COVID-19 pandemic in ECOWAS region?	Africa – West African region	• Capacity building to policy actors on the synthesis of recent data, development of guides and policy briefs
Hendy et al, 2021 [31]	Mathematical modelling to inform New Zealand's COVID-19 response	New Zealand	• Describes how the Stochastic model was used to compare the effects of various interventions in reducing the spread of the virus and to estimate the probability of elimination
Hillmer et al, 2021 [33]	Ontario's COVID-19 Modelling Consensus Table: mobilising scientific expertise to support pandemic response	Ontario – East Central Canada	• COVID-19 Modelling Consensus Table (MCT) is a partnership between the province and academic modellers and consists of multiple experts, health system leaders, and senior decision-makers.
Aguas et al., 2020 [27]	Modelling the COVID-19 pandemic in context: an international participatory approach	Several	• Describes the creation of a participatory modelling approach platform, the COVID-19 Modelling (CoMo) Consortium model, and illustrates some of its use cases
Teerawattananon Et al.,2022 [34]	Recalibrating the notion of modelling for policymaking during pandemics	Several	• Use fitness-for-purpose flowchart and reporting standards trajectory to address the challenges in using models for policymaking.<br>• Adopting such tools can provide a strong justification for increased funding essential for preventing and responding to public health emergencies.
Gombos et al., 2020 [32]	Translating Scientific Knowledge to Government Decision Makers Has Crucial Importance in the Management of the COVID-19 Pandemic	Hungary	• Formation of a multidisciplinary research team performed a large amount of scientific data analysis and mathematical and socioeconomic modelling of the COVID-19 epidemic
