# Supplementary material for: Lessons learned from COVID-19 modelling efforts for policy decision-making in lower- and middle-income countries
Source: BMJ Glob Health. 2024 Nov 8;9(11):e015247. doi: 10.1136/bmjgh-2024-015247 (PMC11552008; doi:10.1136/bmjgh-2024-015247)
Supplement: online supplemental file 5 [file bmjgh-9-11-s005.pdf]

Supplementary File S5 Interview guides

Interview guide for researchers (English)

Interviewee code	
Country code	
Organisation code	
Date	
Position/Role	
Age group	
Gender	
Period I have worked in this role.	
Involvement in Technical Working Group (TWG)	

(After introducing yourself, explaining the purpose of the interview, seeking consent, and finding a setting and time that will make the interviewee comfortable, below is a list of topics to be discussed. The topic guide will remain flexible concerning what is relevant to participants)

- Can you tell me what your role(s) have been as an epidemiologist/statistician/modeler in this organisation, how long have you served in this capacity? Have your responsibilities changed in response to the ongoing COVID-19 pandemic? If yes, in what ways? How has this affected your morale/motivation?
  (Probe: type of modelling done: mathematical, economic, etc.; specify which disease areas they have worked in; identify if roles have changed due to demand from the pandemic?)

- What does it mean for you to be a scientist working in the public eye on COVID-19?
  (Probe: has the experience been positive or negative? In what ways?)

- Have you been involved in presenting evidence to inform policy? How has your experience in being part of this?
  (Probe: in what capacity? policy briefs; TWG meetings; news briefings; other?)

- What institutional/group changes, if any, have you had to make to accommodate evidence demands/support knowledge sharing?

- How have you found collaborating with government and policy organisations? How have these collaborations evolved, especially with the ongoing pandemic?

  (Probe: What impact has this experience had on your relationship with policymakers?)

- Have you been part of collaborative efforts with other modelling groups? Which ones? How has the experience been?

- Are you aware of any of the formal knowledge translation methods? Have you ever used any of them? Specify which?
  (Probe: -targeted dissemination; involving users in the research process; developing networks between researchers and users; use of knowledge brokers)
  (Probe: what capacity needs have you experienced? Has your capacity to communicate evidence improved?)

- Describe which approaches you/your organisation use to share your research findings with policymakers: i) outside the pandemic; ii) during the pandemic?
  (Probe: long-form reports; short country reports; policy/issue briefs; interactive websites; webinars; newspapers; blogs; websites etc.) ask them to specify where a combination has been used.

- Have policymakers ever approached you/your organisation to request data, or do you approach them to share your findings? Have you been part of a technical working group/advisory board?
  (Probe: how has this changed with the pandemic? What has been your experience in being part of a TWG/advisory board?)

- What has been your experience trying to make your research findings of complex mathematical models more palatable to lay people?
  (Probe: have you had any specific training in science communication? Does your organisation offer this support? Has this changed with the pandemic)?
- How have you been involved in presenting scientific evidence to the public, and how have you found this?
  (Probes: How has your role in informing the public about COVID-19 evolved?)
- How do you feel about how the press covers your scientific contributions?
- How do you feel about sharing your scientific evidence on social media?
- How do you feel about how the government presents (your) scientific evidence to the public?
- What kind of reactions have you received from the public? Have you received disturbing reactions or threats? How did you deal with this?
- How has your involvement in COVID-19 research and advising policy/government affected your professional life?
- How do your COVID-19 roles and responsibilities impact your existing roles?
- How has your involvement in COVID-19 research and advising policy/government affected your personal life? How have you dealt with that?
- What have you learned from your experience of informing COVID policy?

(Probe: What do you think are the most important lessons from this experience? What can be done to improve things?)

- If a new pandemic breaks out in the future, what should be done differently regarding sharing information and bringing scientists and policymakers together?
- Do you have any additional remarks?
- Is there something you think we didn't cover relevant to this issue/topic?
- Is there someone else you think we should talk to?

(Thank the participant for their time and find out if they are okay with being followed up later to clarify things and to share the overall findings and invite them to be part of the learning sessions.)

Interview guide for policymakers (English)

Interviewee code	
Country code	
Organisation code	
Date	
Position/Role	
Age group	
Gender	
Period I have worked in this role.	
Involvement in Technical Working Group (TWG)	

(After introducing yourself, explaining the purpose of the interview, seeking consent, and finding a setting and time that will make the interviewee comfortable, below is a list of topics to be discussed. The topic guide will remain flexible concerning what is relevant to participants)

- Can you tell me what your role(s) have been as a policy maker, how long have you served in this capacity?

(Probes: Have your responsibilities changed in response to the ongoing COVID-19 pandemic? If yes, in what ways? How has this affected your morale/motivation?)

- What does it mean for you to be a policymaker working in the public eye on COVID-19?

(Probe: has the experience been positive or negative? In what ways?)

- Have you been involved in using evidence to inform policy? How has your experience in being part of this?

(Probe: in what capacity? Reading policy briefs/manuscripts; TWG meetings; news briefings; other?)

- How have you found collaborating with research organisations? How have these collaborations evolved, especially with the ongoing pandemic?

(Probe: What impact has this experience had on your relationship with researchers?)

- Describe which approaches researchers use to share your research findings with you: i) outside the pandemic; ii) during the pandemic?

(Probe: Probe: Have these approaches increased your accessibility to evidence?)

- Have you, as a policy maker, ever approached a researcher/research organisation to request data, or do they approach you to share their findings? Have you been part of a technical working group/advisory board?

(Probe: how has this changed with the pandemic? What has been your experience in being part of a TWG/advisory board?)

- What has been your experience in trying to understand/make sense of research findings of complex mathematical models?
  (Probe: have you had any specific training in science communication? Does your institution offer this support? Has this changed with the pandemic? Has your capacity to use research evidence improved?)

- How have you assessed the quality of modelling evidence that you use to make decisions?

- Do you intend to continue seeking research evidence to make policy decisions in the future?

- In your opinion, what impact has evidence exchange had on COVID-19 policymaking? Give local examples if available.

- How have you been involved in presenting scientific evidence to the public, and how have you found this?

(Probes: How has your role in informing the public about COVID-19 evolved?)

- How do you feel about how the press covers scientific contributions?

- How do you feel about sharing scientific evidence on social media?

- How do you feel about how scientific evidence is presented to the public?

- What kind of reactions have you received from the public? Have you received disturbing reactions or threats? How did you deal with this?

- How has your involvement in COVID-19 policy decision-making affected your professional life?

- How do your COVID-19 roles and responsibilities impact your existing roles?

- How has your involvement in COVID-19 policy decision-making affected your personal life? How have you dealt with that?

- What have you learned from your experience of generating COVID policies?

(Probe: What do you think are the most important lessons from this experience? What can be done to improve things?)

- If a new pandemic breaks out in the future, what should be done differently regarding sharing information and bringing scientists and policymakers together?

- Do you have any additional remarks?

- Is there something you think we didn't cover relevant to this issue/topic?
- Is there someone else you think we should talk to?

Thank the participant for their time, find out if they are okay with being followed up later to clarify things, share the overall findings, and invite them to be part of the learning sessions.
