# Supplementary material for: Lessons learned from COVID-19 modelling efforts for policy decision-making in lower- and middle-income countries
Source: BMJ Glob Health. 2024 Nov 8;9(11):e015247. doi: 10.1136/bmjgh-2024-015247 (PMC11552008; doi:10.1136/bmjgh-2024-015247)
Supplement: online supplemental file 6 [file bmjgh-9-11-s006.pdf]

Supplementary File S6 Codebook

Name	Description
**1. Knowledge Creation (Knowledge creation processes)**	
a. Knowledge co-production	Narrations on knowledge co-production processes, i.e., where researchers and policymakers report on collaborative meetings to discuss questions that might have led to the need for modelling and use of modelling evidence
b. knowledge synthesis	Narrations on knowledge synthesis approaches utilised, including literature or document reviews/reports.
c. modelling	Narrations on the types of modelling done by the researchers
**2. Strategies/ activities of Knowledge Transfer (K**nowledge transfer/translation methods utilised)	
a. Knowledge co-production	Knowledge co-production [knowledge production as well]
b. embeddedness	Narrations on scenarios/instances of researcher embeddedness with policymakers
c. Knowledge brokers	Narrations on the use of knowledge brokers and their roles
d. Interviews<br>(Print & electronic interviews)	Narrations on the use of newspapers and any other printed material as an approach for KT
f. policy briefs	Narrations on the use of Policy briefs/reports as a method of KT between researchers and Policymakers
g. infographics	Narrations on the use of infographics as a method of KT between researchers and Policymakers
h. dashboards	Narrations on the use of Websites, dashboards, or any other electronic method on KT
i. presentations	Narrations where researchers made online/physical PowerPoint presentations to policymakers
j. policy engagement workshops	Narrations on the use of policy engagement workshops as means of KT between researchers and Policymakers
k. report	Narrations on the use of reports method of KT between researchers and Policymakers
**3. Facilitators (of successful knowledge translation)**	

Name	Description
a. Relationship– (through collaborations/partnerships with government and institutions)	Narrations where relationships, whether previously established/existing or newly created, between researchers/modellers, Government, and other institutions helped in evidence generation (availability/access to data) and translation of evidence to policy.
b. Trust	Narrations that describe the importance of trust and trust building between researchers/modellers and policymakers. And the implication on evidence uptake/use.
c. credibility	Narrations where the credibility of an institution is mentioned
d. capacity building	Narrations on the need for training researchers/modellers/media on science communication
e. media	Narrations on the use of media (social media, press, TV, newspaper) in making the process of KT a success
**4. Barriers on KT (Barriers to successful Knowledge Transfer/translation)**	
a. Communication skills	Lack of capacity (in communication skills) - Narrations on lack of good communication skills among researchers
b. Misinterpretation	Narrations on the policymakers lacking knowledge to understand modelling work leading to wrong interpretation, or description of the government presentation of results as misrepresented
c. Timeliness	Narrations on the strict timelines that were set by policymakers for researchers to respond to the questions raised by the policymakers or even give feedback to the policymakers on issues raised
d. Relationships	Narrations on the difficulty of engaging the government officials/policymakers due to the lack of existing relationships
e. Organizational bureaucracy	Narrations on organisational bureaucracy (Delays in approvals/Challenges in engagement in the research process due to delays from the government
f. Knowledge brokers	Narrations on the challenges of using knowledge brokers in KT
g. Media	Narrations on the challenge of using the media (social media, print media, TV, Radio) on KT
h. quality data	Narrations on Concerns about poor quality of data
**5. Lessons learnt or recommendations**	

Name	Description
a. Personnel	Narrations that describe capacity building on modelling, communication skills, and any other mode of capacity building of researchers/policymakers to improve KT
b. behaviour change	Narrations on behaviour change- e.g., policymakers can listen to researchers and scientists.
c. timeliness	Narrations on the importance of timeliness when engaging stakeholders/policymakers in ensuring that your topic is timely and relevant, as well as issues of concern at that moment
d. social media	Narrations on how to leverage social media (media generally, print, TV, Radio) as a method of KT
e. exit meetings	Narrations on the importance of conducting brief feedback meetings with people as they wait for the comprehensive report
f. continuous process	Narrations that describe the KT as a continuous process, through a constant engagement between the researchers and policymakers and not a one-time thing
g. collaborations<br>(Including Institutional collaborations)	Narrations described the importance of working together between different entities (researchers, modellers, policymakers).
h. coordination	Narrations on explicit coordination mechanisms within and between collaborating and non-collaborating organisations and government
**Recommendations**	
government and partners	Narrations on the partnerships between governments, partners, and other research institutions. Including access and use of local people/organisations as an essential aspect during pandemics
**6. Knowledge translation infrastructure (infrastructure needed for successful knowledge translation)**	
a. Knowledge translation frameworks	Narrations on the importance of having a KT framework or strategy that can be adapted to support the KT process
b. Modelling expertise	Narrations on the importance of the capacity building of personnel, modellers, communication teams, and policymakers' know-how on science communication
c. Modelling software	Narrations on the importance of having modelling software available for use by modellers
d. Accuracy of the evidence	Narrations on the accuracy of the evidence and the need for explicit communication on any assumptions made

Name	Description
e. Media	Narrations on the importance of the use of media (social media, print media, TV, Radio) as a KT approach
f. Power imbalance	Any narrations on power plays, e.g., Power dynamics between international and local players
g. funding	Narrations on the need/availability/or lack of funding to support modelling
**7. Best Practices (perceived)**	
a. National taskforce on COVID-19 response	Narrations on the existence/creation of a COVID-19 Taskforce to facilitate the link between policymakers and researchers to ease the KT process
b. Review of reports	Narrations on conducting a review of reports among the policymakers and researchers/modellers before a formal feedback meeting
