# Supplementary material for: Lessons learned from COVID-19 modelling efforts for policy decision-making in lower- and middle-income countries
Source: BMJ Glob Health. 2024 Nov 8;9(11):e015247. doi: 10.1136/bmjgh-2024-015247 (PMC11552008; doi:10.1136/bmjgh-2024-015247)
Supplement: online supplemental file 7 [file bmjgh-9-11-s007.pdf]

Supplementary File S7 Snapshot of the Thematic Analytic Framework matrix

Theme	Definition	Sub theme	Data	Narrative
Capacity	Capacity includes technical skills to conduct both mathematical modelling and KT. It also includes capacity to use model outputs to make policy decisions.	Modelling experti	*if we had modellers, if we had real time modellers, we would have information to guide the policymakers because they wanted to have geographic specific interventions for Covid 19 response for example, but it is the models that inform them and so I want to tell you that we as a country, we kept waiting for CDC, USCDC to be able to guide but also inform us of which model and what is likely to work out where. (Policymaker 03, Africa)*<br><br>*We have shortage of modelling expertise (Policymaker 03)*<br><br>*I would not be the right person to talk about the quality of modelling, but however I can comment on the fact that this pandemic allowed us to use a modelling in terms of involvement in the pandemic and I think this will be a great avenue for us to explore future outbreaks, future pandemics or epidemics that will happen in our country because now we do have or we have sort of piloted on how modelling would assist in terms of decision making, in terms of intervention that needs to be done to prevent more mortality from these diseases. (Policymaker, 01 - Africa)*<br><br>*We had very talented, um, advisors with us, a few of them, and they, they were very helpful to, you know, trying to get all the, uh, results of the modeling from different groups and try to make it integrated and come up with the, the summaries of, you know, what for each task that we've been asked to do, everyone tried to use their own model to produce some result. Sometimes results agree, sometimes results, completely different. And these advisory people trying to help guiding us into the decision as a, as a group so that, you know, the modelers can, uh, work together peacefully and also benefit from each other in that modeling exercise. It, I think it was quite a challenging job to do, you know when you listen to three different models and you have to try to make sense of all of them and come up with a conclusion every time. (Researcher 9)*	Researchers & policy makers underscored the importance of having a pool of modellers who are available to generate locally relevant mathematical models. To make decisions tailored to the local context, policy makers need model outputs that reflect their current situation. Modellers were concerned about relying on partners and other non-local organisations for their modelling needs both in terms of the type of model utillised and the relevance of the outputs to their setting.
		**Training for KT**	*The issue of science communication is a very big problem I am telling you in Uganda here because researchers, no the scientists they openly tell you that for us we don't speak much, it is those politicians who speak much. I am not trained to speak to media houses for example, so they want to interview him and he would tell you that is rumours, I don't want to speak to those guys, they are wasting my time. (Policymaker 03)*<br><br>*I think knowledge translation should be spread across different programmes. Even modellers need to be trained on knowledge translation and people who are in knowledge translation should be trained on what modelling is all about. So that even at the institutional level, we have modellers speaking with knowledge translators is… and communication department, a language that is easy for the public to understand. (Researcher 01)*<br><br>*I think it's… it's… uum… it's faster, more efficient uum… and more impactful if modellers are trained in how to communicate and speak the language of the policymaker (Researcher 07)*	
