# Supplementary material for: Lessons learned from COVID-19 modelling efforts for policy decision-making in lower- and middle-income countries
Source: BMJ Glob Health. 2024 Nov 8;9(11):e015247. doi: 10.1136/bmjgh-2024-015247 (PMC11552008; doi:10.1136/bmjgh-2024-015247)
Supplement: online supplemental file 8 [file bmjgh-9-11-s008.pdf]

Supplementary File S8 Good Reporting of A Mixed Methods Study (GRAMMS) checklist

Guideline	Section: page
Describe the justification for using a mixed methods approach to the research question	Methods- under study design pg 6
Describe the design in terms of the purpose, priority and sequence of methods	Methods- procedures pg. 7-9
Describe each method in terms of sampling, data collection and analysis	Procedures pg 7-9
Describe where integration has occurred, how it has occurred and who has participated in it	Design and analysis: pg. 7-9
Describe any limitation of one method associated with the present of the other method	Discussion pg.22
Describe any insights gained from mixing or integrating methods	Discussion: pg. 22

O'Cathain A, Murphy E, Nicholl J. The quality of mixed methods studies in health services research. J Health Serv Res Policy. 2008;13: 92-98.
